# Psychometric properties of the wellbeing literacy 6-item scale in Chinese military academy cadets

**DOI:** 10.3389/fpsyg.2024.1293845

**Published:** 2024-03-01

**Authors:** Zhen Jia, Fangfang Zheng, Feifei Wang, Guoyu Yang

**Affiliations:** ^1^Department of Developmental Psychology of Armyman, School of Psychology, Army Medical University, Chongqing, China; ^2^School of Basic Medicine, Army Medical University, Chongqing, China

**Keywords:** well-being literacy, Chinese version of Well-lit 6, reliability, validity, military academy cadets

## Abstract

**Background:**

Positive psychology is a vibrant field of study, and conceptualizations of the components of well-being have received a great deal of attention from researchers. The study of well-being literacy thus provides an innovative perspective for enhancing and sustaining individuals’ experiences of well-being.

**Objective:**

This study aimed to examine the psychometric properties of the wellbeing literacy 6-item (Well-Lit 6) scale in Chinese military academy cadets.

**Methods:**

A total of 3,218 undergraduate students from five military academies in China were recruited to complete questionnaires online.

**Results:**

(1) The items of the scale showed high discrimination; (2) The alpha coefficient of the scale was 0.986 and the split-half reliability was 0.981, indicating high homogeneous reliability and split-half reliability; (3) The scale model fitted well and displayed structural validity; (4) The correlation between well-being literacy and related indicators was significant, and the calibration correlation and convergent-discriminant validity of the scale were high; (5) After gradually adding demographic variables, known predictors factors and well-being literacy, the *∆R*^2^ for subjective well-being, life satisfaction, depression, and anxiety ranged from 0.036 to 0.067, 0.184 to 0.340, and 0.009 to 0.017, respectively, showing high incremental validity; (6) the total well-being literacy scores differed significantly by gender, grade, and parenting style.

**Conclusion:**

The Chinese version of the Well-Lit 6 is reliable and valid in predicting and accessing the subjective well-being, life satisfaction, emotion regulation, and psychological resilience of Chinese military academy cadets.

## 1 Introduction

Positive psychology is a vibrant field of study, and conceptualizations of the components of well-being have received a great deal of attention from researchers (Wang et al., 2023). According to literature, subjective well-being (SWB), as a complex, multidimensional state, encompasses individuals’ personal judgments of their life satisfaction, guided by internal and societal standards, and characterized by a dominance of positive emotions (Diener, 2000). Extensive research suggests that language plays a critical role in expressing, integrating, and comparing positive emotions, subsequently influencing emotional regulation. The acquisition of language skills is vital for individuals to effectively navigate and regulate their emotions in social contexts (Saarni, 1999). Furthermore, emotional regulation has been found to significantly improve subjective well-being. Building upon these findings, Lindsay G. Oades introduced the concept of “wellbeing literacy” and further delved into its implications (Oades, 2017). Well-being literacy refers to the vocabulary, knowledge and language skills that are intentionally used to maintain or improve the well-being of oneself, others and the world (Oades, 2017). It is about how and why people use language in their everyday lives, and how they use language to create experiences of well-being for themselves or others (Oades et al., 2020). In contrast to the more subjective and transient feeling of “happiness,” the concept of “well-being literacy” highlights the ability to use language to create a stable, sustained sense of well-being. In 2021, Oades presented his latest research at the 5^th^ International Conference on Positive Psychology in China, and argued that well-being literacy is the intermediary or mediator between the internal and external environment and the experience of well-being, i.e., the higher the well-being literacy, the stronger and more sustained the experience of well-being in the same internal and external environment (Oades et al., 2021a,b). The study of well-being literacy thus provides an innovative perspective for enhancing and sustaining individuals’ experiences of well-being.

To conceptualize and measure wellbeing literacy, Oades presented five components in his capability model of wellbeing literacy (Oades et al., 2020, 2021a,b), involving (1) vocabulary and knowledge about wellbeing (words and basic facts), (2) comprehension of multimodal text related to wellbeing (reading, listening and viewing), (3) composition of multimodal text related to wellbeing including writing, creating, and speaking, meaning that individuals could verbally express their feelings to others, social media posts, writing blogs or singing songs, (4) context sensitivity, referring to the awareness of differences across contexts and adaptive use of language to fit the relevant context, and (5) intentionality – an ongoing desire to improve the wellbeing of self, others or the world.

Hanchao Hou et al. developed the Wellbeing Literacy 6 Item Scale (Well-Lit 6) based on the theoretical structure of well-being literacy, and proved that the scale has reliability and validity in both foreign students and non-student samples (Hou et al., 2021). There is limited literature available on wellbeing literacy apart from the work by Mahmic Sylvana and Kern Peggy, who focus on empowering families through a system-informed approach. Moreover, the existing research on wellbeing literacy is often confined to specific language cultures and populations, such as English (Mahmic and Kern, 2021). Establishing measurement invariance, or equivalence of measurement structures across groups, is critical to ensure that scores across groups have the same meaning (van de Schoot et al., 2012). Thus, the study of military personnel as a unique demographic is essential, as they embody a distinct subgroup with sociocultural and educational experiences not paralleled in civilian education systems (Ghosh et al., 2022). Military academies in China represent a unique sociocultural and educational environment, with distinct values, discipline, and stressors not found in civilian education. These cadets experience a dual identity as “soldier” and “student,” which subjects them to a compounded set of responsibilities and pressures (Myers and Bechtel, 2004). Given the exhaustive academic and military training curriculum, strict performance criteria, and rigorous discipline within these military schools, cadets face an increased risk of developing psychological and behavioral issues (Gold and Friedman, 2000; Perez Ade and Bensenor, 2015). This is supported by Zhao’s research (Zhao et al., 2023), which found that among 1,430 Chinese soldiers, 162 soldiers (11.33%) presented with mental disorders, indicating a relatively high prevalence. Further studies suggest that happiness not only can improve military performance (Lester et al., 2022), but also plays a significant role in dominating and determining the quality of mental health, with a bidirectional influence between mental health and overall well-being (Diener et al., 2017). Thus, it is essential to accurately evaluate well-being literacy within Chinese military academy cadets. Adequate assessment can yield significant health advantages and holds key implications for future enhancements of cadet life satisfaction and psychological health. This highlights the need for targeted research within this unique educational setting, aiming to develop tailored strategies to support the well-being and mental health of these individuals.

This experiment aims to assess the reliability and validity of the Chinese version of the Well-Lit 6-item scale among Chinese military academy cadets, test the uniqueness of the theoretical construct of “well-being literacy,” and investigate the characteristics of it among Chinese military academy cadets, to analyze the scale items, to test the homogeneous reliability and split-half reliability; to test the construct validity through exploratory factor analysis. Positive indicators (e.g., subjective well-being, life satisfaction) and negative indicators (e.g., depression, anxiety) that have been shown to be related to wellbeing literacy were included to verify the validity of the correlational and convergent-discriminant validity of the validity scales (Oades et al., 2020; Hou et al., 2021); a large number of previous studies have confirmed that emotion regulation and psychological resilience are effective predictors of subjective well-being (Jin and Wang, 2016; St-Louis et al., 2020), life satisfaction (Li et al., 2017; Nam et al., 2017), depressive states (Whitehead and Suveg, 2016; Haghighi and Gerber, 2019) and anxiety states (Haghighi and Gerber, 2019; Cook and Newins, 2021), we used this to test the incremental validity of the scale in explaining subjective well-being, life satisfaction, depressive states and anxiety states on the basis of emotion regulation and resilience indicators; finally, we explored characteristics of the well-being literacy among Chinese military academy cadets.

## 2 Methods

### 2.1 Participants and procedure

A total of 3,218 cadets were recruited for this study in January 2023 from five military academies using convenience sampling. All participants completed the survey by scanning the QR code of the questionnaire in the classroom, which was guided by a psychology faculty member on-site. The survey was supported by the Questionnaire Star web platform, which is available at www.wjx.cn. Their informed consent and questionnaires were obtained with an effective rate of 100%. There were 2,883 male cadets (89.6%) and 335 female cadets (10.4%); 860 freshmen (26.7%), 1,069 sophomores (33.2%), 512 juniors (15.9%) and 777 seniors (24.2%); 1,406 singletons (43.7%), 1,812 non-singletons (56.3%); 2,929 from two-parents families (91.0%), 289 from single-parent or other families (9.0%); 319 (9.9%) participants grew up in autocratic family parenting style, 527 (16.4%) permissive and 2,372 (73.7%) democratic; aged 16 to 31, mean age (20.67 ± 2.07).

### 2.2 Measurements

#### 2.2.1 The Chinese version of the Well-lit 6 scale

The Well-Lit 6 was developed from the Well-Lit theory and was based on the Australian Curriculum, Assessment and Reporting Authority (ACARA)’s definition of the term “literacy.” The scale is rated on a 7-point Likert scale from 1 “strongly disagree” to 7 “strongly agree” and shows high internal consistency in the foreign sample (students: *α* = 0.84, staff: *α* = 0.91, parents: *α* = 0.91; Hou et al., 2021). Our first author contacted the scale developers, Hanchao Hou and Lindsay G. Oades via email and obtained their permission. The scale was later translated into Chinese by the original authors in collaboration with Jinting Liu’s team at Shenzhen University.

#### 2.2.2 Self-made basic personal information questionnaire

This scale includes information on grade, gender, family structure, parenting style and whether they are well adjusted to school environment.

#### 2.2.3 The general well-being schedule (GWB)

The GWB, developed by the National Center for Health Statistics, was used to assess participants’ subjective well-being (Fazio and Stat, 1977). The Chinese revised version of the scale includes 6 dimensions (Liu, 1996), involving energy, control over emotions and behavior, relaxation and tension (reverse scoring), depressed or happy feelings, concerns about health and satisfaction and interest in life, with a total of 18 items, rated on a scale of 1 to 5 for questions 2, 5 to 7, 1 to 6 for questions 1, 3, 4, 8 to 14 and 1 to 18 for questions 15 to 18. Questions 1, 3, 4, 8–14 are rated 1–6, questions 15–18 are rated 1–10 and the total score is 120. The higher the scale score, the better the subjective well-being. The internal consistency coefficient of the scale is 0.91–0.95, and the retest reliability is 0.85. The scale has high reliability and validity.

#### 2.2.4 Life satisfaction scales applicable to college students (CSLSS)

The CSLSS, developed by Yuzhong Wang and Songhe Shi, was used to assess the life satisfaction of university students (Wang and Shi, 2003). The scale is divided into two dimensions, with the first five questions involving objective satisfaction (physical health, study, financial situation, appearance and performance, and interpersonal relationships) and the sixth question concerning subjective satisfaction, i.e., one’s overall satisfaction with life. The scale is rated on a 7-point scale, from 1 “very dissatisfied” to 7 “very satisfied.” The final score got via adding up the scores of the first 5 questions and then dividing by 5 is taken as an objective satisfaction score, and question 6 as a subjective satisfaction score. The two were then added together to get a total personal life satisfaction score. The Cronbach’s *α* coefficient for this scale is 0.716. The scale has been proven to have high validity and theoretical validity.

#### 2.2.5 Patient health questionnaire depression scale (PHQ-9)

The Depression Screening Scale (PHQ-9) proposed by Kroenke et al. (2001) is also used in this study. PHQ-9 is a self-assessment tool to screen for depression, using the American Diagnostic and Statistical Manual of Mental Disorders, 4th edition (DSM-IV) as a reference standard and contains 9 items. It is adapted to assess the subject’s feelings over the past two weeks, with 4 options for each item, scoring from 0 to 3, with a total score range of 0 to 27, and higher scores indicating more severe depressive symptoms.

#### 2.2.6 Generalized anxiety disorder 7-item (GAD-7)

The Generalized Anxiety Disorder 7-item (GAD-7) is a quantitative assessment standard suggested by the Diagnostic and Statistical Manual of Mental Disorders, Fifth Edition (DSM-V), which was published by the American Psychiatric Association (APA) in May 2013 and was nationally recommended by the Psychiatric Branch of the Chinese Medical Association on June 8, 2015. These two scales are simple, highly operational, and have been validated by domestic and international studies (Spitzer et al., 2006), and can be used to screen population in the community and specific groups (Bouchoucha et al., 2013), assisting physicians to rapidly screen anxious and depressed patients and to monitor changes in their conditions.

#### 2.2.7 Emotion regulation questionnaire (ERQ)

The ERQ developed by Gross was used to assess the emotion regulation strategies of military academy cadets. It contains two dimensions, with the cognitive reappraisal dimension containing 6 items and expressive inhibition dimension involving 4, and is scored on a 7-point Likert scale, with higher scores meaning that the subject uses that emotion regulation strategy more frequently (Gross and John, 2003). The scale has a test–retest reliability coefficient of 0.82 and a Cronbach’s *α* coefficient of 0.85, and for the Expressive Inhibition dimension, the test–retest reliability is 0.79 and Cronbach’s *α* coefficient is 0.77.

#### 2.2.8 Connor-Davidson resilience scale (CD-RISC-10)

This scale is a simplification of the 25-item CD-RISC by Campbell-Sills and Stein (2007), and the Chinese version was revised by Zhang et al. (2018). It contains 10 items and is scored on a 5-point Likert scale, i.e., 1 for “never,” 2 for “rarely,” 3 for “sometimes,” 4 for “often” and 5 for “always.” The total score of the scale is a direct sum of the scores of each item, with higher scores indicating greater psychological resilience.

### 2.3 Ethics approval and consent to participate

This study was conducted in accordance with the Declaration of Helsinki. Ethical approval was obtained from the Ethics Committee of Army Medical University (NO. [2020] 028–02). Each participant was informed in a written consent under the headline of the questionnaire that participation in the study was voluntary and that they could withdraw from the study at any time even after voluntarily participating without offering any reason. They were also informed that the survey was anonymous, and were assured that personal information would not be disclosed.

### 2.4 Statistical processing

SPSS 25.0 and AMOS 22.0 were used for statistical analysis. Count data were expressed as cases or percentages; measurement data were expressed as ( $\bar{x}$ *±s*); reliability of the scale was tested by Cronbach’s *α* and split-half reliability; validity of the scale was evaluated by convergent validity, structural validity (exploratory factor analysis and validation factor analysis were conducted by SPSS 25.0 and AMOS 22.0 respectively), and incremental validity. Independent samples *t*-test and one-way ANOVA were used to compare demographic differences in indicators of well-being literacy at a test level of *α* = 0.05.

## 3 Results

### 3.1 Item analysis

The correlation coefficients between each item and the total score of the scale ranged from 0.95 to 0.97 (*p* < 0.001) (see Table 1), and 27% of the subjects whose well-being literacy total score ranked in the front and behind were selected as the high group and the low group, and independent sample *t*-tests were conducted in both groups. The results showed that there were significant differences between the high and low groups (*p* < 0.001) which proved high discrimination of the scale.

**Table 1:** Results of the item analysis and exploratory factor analysis of the Chinese Well-Lit 6 scale.

Item	r	t	Factor loading	Degree of commonality
1. I have many words I can think of to communicate about wellbeing	0.96^*^	−61.95^*^	0.96	0.92
2. I know a lot about wellbeing	0.97^*^	−62.82^*^	0.97	0.94
3. I know how to improve my wellbeing	0.97^*^	−58.95^*^	0.97	0.93
4. I have the skills to understand information about wellbeing	0.97^*^	−54.06^*^	0.98	0.95
5. I have the skills to express myself about wellbeing	0.97^*^	−55.00^*^	0.97	0.95
6. I can communicate about wellbeing in multiple ways (e.g., writing, listening, drawing) to suit the needs of my audience	0.95^*^	−64.52^*^	0.95	0.90

*p < 0.001.

### 3.2 Reliability

Reliability analysis was conducted using the overall sample to test for homogeneous reliability and split-half reliability. The Cronbach’s *α* was 0.973 (see Table 2), and the Cronbach’s *α* obtained by removing any of the items was less than this coefficient, indicating homogeneous reliability of the scale; the first three and the last three questions were divided into two parts, and the correlation coefficient and the Gettleman’s half coefficient between the two parts were greater than 0.8, which proved that the scale has high split-half reliability.

**Table 2:** Homogeneous reliability and split-half reliability of the six-item well-being literacy scale in Chinese.

	Item 1	Item 2	Item 3	Item 4	Item 5	Item 6
Cronbach’s *α*			0.986			
Cronbach’s *α* after removing the item	0.983	0.982	0.983	0.982	0.982	0.985
Cronbach’s *α*		0.975^a^			0.973^b^	

a Combine items 1 to 3.

b Combine items 4 to 6.

### 3.3 Validity analysis

#### 3.3.1 Structural validity

##### 3.3.1.1 Exploratory factor analysis

The overall sample was randomly divided into two parts and 1,609 of the valid samples were used for exploratory factor analysis. The results showed that the KMO was 0.929 and the Bartlett’s spherical test result was = 18032.933 (*p* < 0.001), indicating that the data were suitable for exploratory factor analysis, and the factors were extracted for orthogonal rotation using principal component analysis. The results showed (see Table 1) that the Chinese version of the Well-Lit 6 had all questions loaded on the same factor, explained 93.01% of the variance, and the minimum value of commonality is 0.90 and the maximum value is 0.95.

##### 3.3.1.2 Confirmatory factor analysis

**Figure 1:**
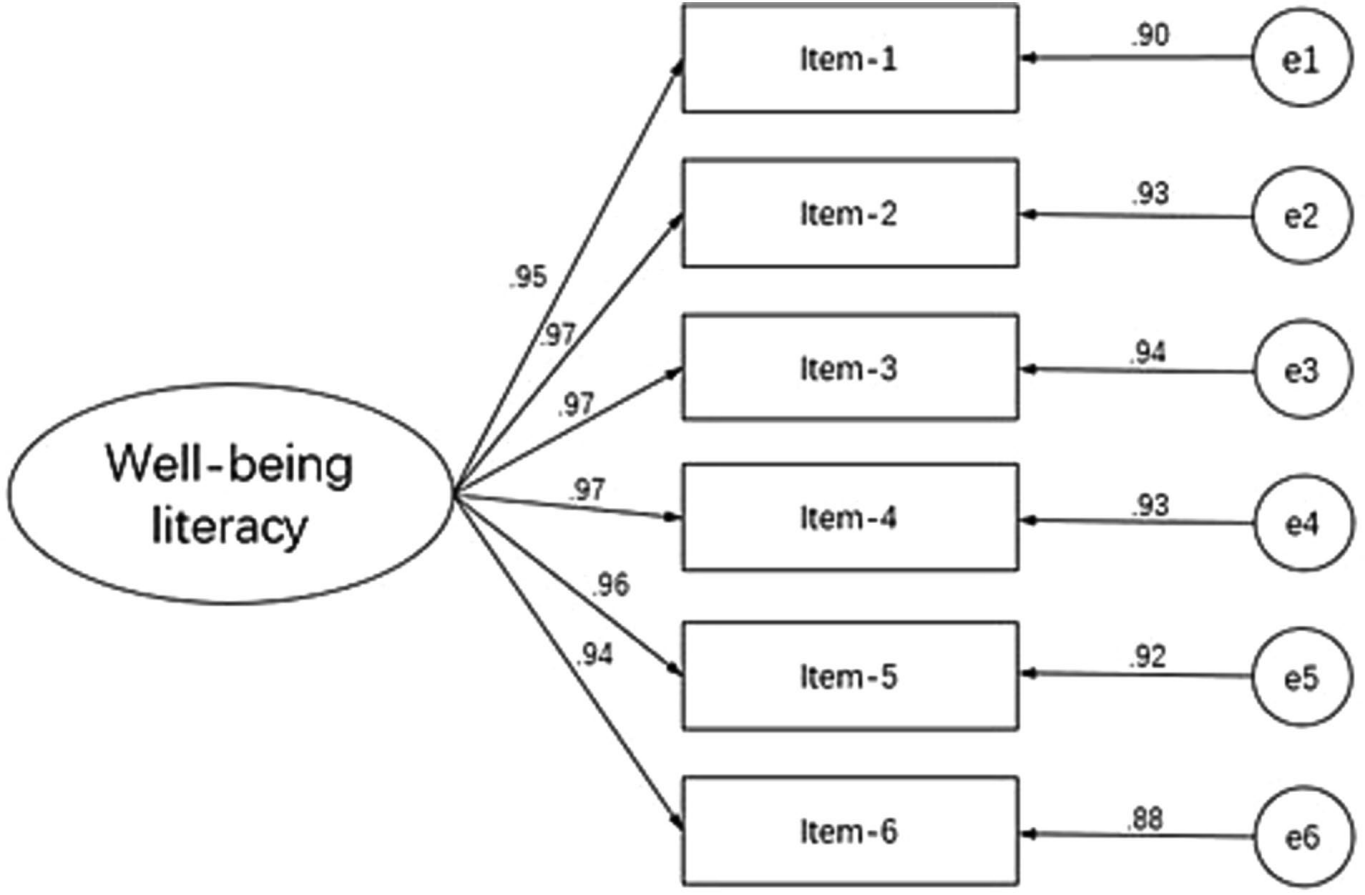
Fitting index of the wellbeing literacy theory.

Confirmatory factor analysis was performed on the remaining 1,609 valid samples by using the software AMOS22.0 to assess the internal structure of the Chinese version of the Well-being Literacy 6-item (Well-Lit 6) scale (see Table 3 and Figure 1). The result indicates that this scale has good structural validity and the question items can effectively reflect the indicators of well-being literacy.

**Table 3:** Validated factor analysis model fit indices.

𝜒^2^/df	NFI	CFI	GFI	TLI	RFI	RMSEA	SRMR
6.873	0.998	0.998	0.993	0.995	0.995	0.060	0.002

#### 3.3.2 Correlation validity of the calibration scale

The well-being literacy of the overall sample was compared to theoretically relevant indicators. The results (see Table 4) showed that well-being literacy was significantly and positively correlated with subjective well-being, life satisfaction, emotion regulation, and psychological resilience (*p* < 0.01) and significantly negatively correlated with depression, anxiety, and expression inhibition (*p* < 0.01). This indicates high calibrated correlation validity of the scale.

**Table 4:** Correlations between well-being literacy and the indicators.

		$\bar{x}$	s	1	2	3	4	5	6	7
1	Wellbeing literacy	34.22	9.76							
2	Subjective well-being	84.82	15. 36	0.280^*^						
3	Life satisfaction	30.33	5.55	0.276^*^	0.570^*^					
4	Depression	4.58	4.95	−0.224^*^	−0.674^*^	−0.539^*^				
5	Anxiety	2.98	4.10	−0.223^*^	−0.650^*^	−0.499^*^	0.866^*^			
6	Emotion regulation	48.39	10.85	0.166^*^	0.209^*^	0.227^*^	−0.139^*^	−0.114^*^		
7	Psychological resilience	30.15	8.25	0.273^*^	0.619^*^	0.523^*^	−0.475^*^	−0.448^*^	0.444^*^	

*p < 0.01.

### 3.4 Convergent-discriminant validity

In the overall sample, there was a significant positive and weak correlation between well-being literacy and emotion regulation (*r* = 0.166, *p <* 0.01) and psychological resilience (*r* = 0.273, *p <* 0.01) (see Table 4), indicating that the scale has relatively high convergent-distinct validity.

### 3.5 Incremental validity

A stratified regression analysis was conducted on the sample, with the dependent variables being subjective well-being, life satisfaction, depression and anxiety, and the demographic variables (gender, grade, singleton or not, family structure, family parenting style), known predictors (emotion regulation, psychological resilience) and well-being literacy added as independent variables. The results (see Table 5) show that the *∆R^2^* of the dependent variables were statistically significant after the addition of the “well-being literacy” variable, which indicates high incremental validity.

**Table 5:** Results of stratified regression analysis.

	Predictive variables	SWB		LS		D		A	
		β	∆*R*^2^	β	∆*R*^2^	β	∆*R*^2^	β	∆*R*^2^
Step 1	Sex^a^	−0.034^*^	0.067^**^	−0.002	0.046^**^	0.030	0.044^**^	0.014	0.036^**^
	Grade^b^	−0.124^**^		−0.061^**^		0.102^**^		0.082^**^	
	Only child^c^	0.044^*^		0.020		−0.016^**^		−0.030	
	Family structure^d^	−0.032		−0.059^*^		0.022		0.014	
	Parenting style^e^	−0.207^**^		−0.190^**^		0.171^**^		0.161^**^	
Step 2	Sex^a^	−0.017	0.340^**^	0.012	0.241^**^	0.016	0.202^**^	0.002	0.184^**^
	Grade^b^	−0.061^**^		−0.007		0.054^*^		0.037^*^	
	Only child^c^	0.028^*^		0.008		−0.004		−0.017	
	Family structure^d^	−0.015		−0.046^*^		0.009		0.001	
	Parenting style^e^	−0.112^**^		−0.108^**^		0.099^**^		0.092^**^	
	Emotion regulation	−0.083^**^		−0.007		0.092^**^		0.107^**^	
	Psychological resilience	0.628^**^		0.505^**^		−0.492^**^		−0.475^**^	
Step 3	Sex^a^		0.012^**^		0.017^**^		0.009^**^	0.006	0.010^**^
	Grade^b^	−0.022							
	Only child^c^	−0.057^**^		0.007		0.020			
	Family structure^d^	0.026							
	Parenting style^e^	−0.016							
	Emotion regulation	−0.105^**^							
	Grade^b^	−0.089^**^		−0.002		0.050^*^		0.033^*^	
	Only child^c^	0.601^**^		0.006		−0.002^**^		−0.015	
	Family structure^d^	0.114^**^		−0.047^*^		0.010		0.002	
	Parenting style^e^			−0.099^**^		0.092^**^		0.085^**^	
	Emotion regulation			−0.014		0.097^**^		0.113^**^	
	Psychological resilience			0.473^**^		−0.469^**^		−0.450^**^	
	Well-being literacy			0.137^**^		−0.099^**^		−0.107^**^	

a 1 = male,2 = female.

b 1 = first year, 2 = second year, 3 = junior year, 4 = senior year.

c 1 = singleton, 2 = non-singleton.

d 1 = two-parent, 2 = single parent and others.

e 1 = democratic, 2 = permissive, 3 = autocratic. β is the standardized coefficient.

*p < 0.05; **p < 0.001.

### 3.6 Characteristics of military academy cadets’ well-being literacy

Independent samples *t*-tests (demographic variables grouped = 2) and one-way ANOVAs (demographic variables grouped >2) were used to explore the well-being literacy and demographic characteristics of the 3,218 undergraduate students (see Tables 6, 7). The results showed that the well-being literacy of female students was significantly higher than that of male; scores of senior students were significantly lower than those of students in other age groups; well-being literacy of students with democratic, permissive and autocratic parenting styles decreased in descending order; there were no significant differences in the well-being of military academy cadets in terms of “family structure” or whether they are the only-child or not.

**Table 6:** Independent sample t-test for well-being literacy (*n* = 3,218).

	Sex		Only-child		Family structure	
	Male (*n* = 2,883)	Female (*n* = 335)	Singleton (*n* = 1,406)	Non-singleton (*n* = 1,812)	Two-parent (*n* = 2,929)	Single parent and other (*n* = 289)
WL ( $\bar{x}$ ±s)	34.09 ± 9.86	35.27 ± 8.84	33.86 ± 10.10	34.49 ± 9.48	34.22 ± 9.78	34.17 ± 9.63
*t*	−2.27^*^		−1.79		−0.090	

注: *p < 0.05.

**Table 7:** One-way ANOVA test for well-being literacy (*n* = 3,218).

	Grade			Parenting style			
	First year (*n* = 860)	Second year (*n* = 1,069)	Junior year (*n* = 512)	Senior year (*n* = 777)	Democratic (*n* = 2,372)	Permissive (*n* = 527)	Autocratic (*n* = 319)
WL ( $\bar{x}$ ±s)	34.91 ± 8.60	34.54 ± 9.82	34.55 ± 10.56	32.77 ± 10.40^a^^b^^c^	34.82 ± 9.75^b^^c^	33.28 ± 9.51^a^^c^	31.56 ± 9.61^a^^b^
*F*	7.76^**^				16.70^**^		

a Statistically significant difference compared to the first set of data.

b Statistically significant difference compared to the second data set.

c Statistically significant difference compared to the third data set.

**p < 0.001.

## 4 Discussion

### 4.1 Reliability and validity of the Well-lit 6 scale

The correlation between the items of the Chinese Well-Lit 6 and the total score ranged from 0.95 to 0.97, and the difference between the scores of the items of the high and low group samples reached a significant level, indicating high discrimination of the scale. The homogeneous reliability is 0.986, the Cronbach’s *α* after deleting any item is between 0.982 and 0.985. The latter is lower than the former. And the Spearman’s split-half reliability is 0.981, indicating high homogeneous reliability and split-half reliability.

The results of the exploratory and validation factor analyses show that the scale has relatively high structural validity and presents a one-dimensional structure, demonstrating that the items on the scale can effectively reflect indicators of well-being; Previous theories and studies have shown that life satisfaction (Krueger and Schkade, 2008), emotion regulation (St-Louis et al., 2020), psychological resilience (Yildirim and Arslan, 2022), psychological capital (Çagis and Yildirim, 2023), personal growth initiative (Green and Yildirim, 2022), self-esteem and gratitude (Murat Yildirim et al., 2019), depression and anxiety (Burns et al., 2011) are all effective predictors of an individual’s subjective well-being. Therefore, in this study, subjective well-being, life satisfaction, emotion regulation, resilience, depression, and anxiety were chosen as the correlation validity scales for well-being literacy. The results showed that the total score of well-being literacy was significantly positively correlated with subjective well-being, life satisfaction, emotion regulation and psychological resilience, with correlation coefficients ranging from 0.166 ~ 0.280, and was significantly negatively correlated with depression and anxiety, with correlation coefficients of −0.224 and − 0.223 respectively, demonstrating that the scale has correlations validity; In addition to being a stable predictor of subjective well-being (Quoidbach et al., 2010; Lee et al., 2013; Hu et al., 2015), emotion regulation and psychological resilience appear to be more stable than life satisfaction, depression, and anxiety, which are descriptive indicators of feelings, similar to well-being literacy (Hou et al., 2021; Oades et al., 2021a,b). Therefore, we chose emotion regulation and psychological resilience as calibration scales for validating the aggregated-discriminant validity of the scale. The results showed that the overall well-being literacy score was only weakly correlated with emotion regulation and psychological resilience, indicating that well-being literacy is both similar to and different from emotion regulation and psychological resilience, and that the scale is of excellent convergent-discriminant validity; The results of the stratified regression analysis showed that wellbeing literacy remained a good predictor of subjective well-being, life satisfaction, depression and anxiety based on demographic variables and known predictors (emotion regulation and psychological resilience), indicating incremental validity. In summary, the Chinese version of the Well-being Literacy 6-Item (Well-Lit 6) scale has high reliability and validity among Chinese Military Academy Cadets, which is consistent with the findings of the relevant literature (Hou et al., 2021).

Furthermore, the reliability measured in this study is significantly higher than that described in the related literature (Hou et al., 2021). The reasons for this is, on the one hand, that the sample size of this study is larger (about 2.5 times the sample size of the original literature) and the results obtained are more reliable. Another reason may be due to the collectivist culture inherent in military academies which emphasizes values such as duty, honor, and the collective good. As Mesquita puts it, in collectivist contexts, an individual’s emotions are closely linked to social value assessment and a sense of belonging with others (Mesquita, 2001). the emotional well-being of cadets is intertwined not just with personal factors, but also with the cohesion and morale of their peers and the larger military community. Maintaining positive relationships and contributing to the group’s welfare becomes integral to their sense of identity and satisfaction. Thus, the collectivist attributes of the military academy culture underpin why this scale shows higher reliability and relevance among Chinese military academy cadets.

### 4.2 The significance of studying the well-being literacy of military cadets

In this study, well-being literacy is significantly and moderately correlated with subjective well-being, life satisfaction, emotion regulation and psychological resilience, indicating that well-being literacy is both similar to and structurally distinct from theoretically relevant indicators, thus demonstrating that well-being literacy remains theoretically unique in Chinese cadets. Meanwhile, well-being is an effective predictor of subjective well-being, life satisfaction, anxiety and depression based on known predictors, which demonstrates the incremental validity of the Chinese version of the Well-Lit 6 scale as well as the relevance of studying the theory of well-being to improve subjective well-being, life satisfaction and reduce anxiety and depression.

This is because well-being literacy is a competence which is believed to be the key to enhancing subjective well-being. Further, the researchers proposed a competency model of well-being literacy, presenting it as a mediator (or moderator) in the experience of well-being. The competency model consists of five components (knowledge of well-being, perception of well-being, expression of well-being, contextual integration and intention to be happy) that interact with internal and external environmental conditions. The level of competence to be achieved depends on the relationship among these factors. If the level of the five components is low, or if the environmental accessibility is low, the competence level may also be low or down to zero (Oades et al., 2021a,b). Military academy cadets not only face heavy academic and training tasks during their time at the academy but also undergo strict military management. They will inevitably encounter stress, setbacks, and other negative events that lead to adverse experiences. In the process of flexibly utilizing well-being knowledge, perceiving well-being, expressing well-being, integrating into contexts, and transforming well-being intentions to resolve these adverse experiences, psychological resilience is naturally enhanced. The stronger an individual’s psychological resilience, the more likely they are to possess higher levels of subjective well-being. In summary, well-being literacy has unique theoretical significance among Chinese military cadets. By enhancing the well-being literacy of the cadets—using knowledge, perception, expression, and intentions related to well-being—it fosters the development of psychological resilience in the face of the pressures and challenges of military academy life, which in turn helps maintain a higher level of subjective well-being.

### 4.3 Features of Chinese military academy cadets’ well-being literacy

In terms of gender, the well-being literacy scores of the female group were significantly higher than those of the male group. This is more consistent with the findings of Zheng et al. (2003) on the well-being of Chinese students, possibly because girls are better than boys at expressing themselves verbally, and girls are also better than boys at perceiving and utilizing emotional experiences. In addition, girls tend to receive more care from the school, administrators, teachers and other students around them, which may also contribute to higher well-being literacy.

From the perspective of grades, the well-being literacy scores of senior students were significantly lower than those of other grades. The reason for this difference may be that graduating students start facing a series of realistic pressures such as thesis writing, graduation assessments and workplace assignment, which in turn lead to a decrease in well-being literacy. This is also partially consistent with the findings of Lu et al. (2013) on the influence of grade on Chinese military academy cadets; therefore, future interventions on the well-being literacy should focus on the senior groups in Chinese academies.

Shek and Liang’s research (Shek and Liang, 2018) indicates an association between parental psychological control and an individual’s subjective well-being. Therefore, one hypothesis in the current study might be that parental upbringing styles exert a certain influence on an individual’s well-being literacy. According to the research findings, the researchers observed that the levels of well-being literacy for participants with democratic, permissive, and autocratic parenting styles decrease in that respective order. This suggests that individuals with a democratic upbringing have more verbal interaction with their family members throughout their development, possess a richer knowledge and vocabulary concerning happiness, and exhibit a stronger ability to perceive and express happiness. In comparison, those raised in permissive parenting styles have less interaction with their family members, which correlates with a comparatively reduced ability to enhance well-being literacy. Furthermore, individuals brought up with an autocratic parenting style often grow up in a submissive environment, leading to limited verbal communication with family members and, consequently, a lack of language-based skills related to well-being literacy. These research outcomes imply that different parenting styles may have an impact on an individual’s level of well-being literacy. A democratic parenting approach may contribute to fostering an individual’s well-being knowledge and capabilities, whereas permissive and autocratic styles might constrain the development of well-being literacy.

Since well-being literacy involves an individual’s ability to understand multimodal texts related to well-being, those who are adept at recognizing contextual differences and accordingly adapt their language to suit the various contexts they encounter, usually have higher well-being literacy.

In the study at hand, family structure was categorized into “two-parent households” and “single-parent and others” with the objective of exploring the potential impact of the familial environment on individual well-being and development. Conventionally, it is believed that two-parent households provide a more supportive and communicatively rich environment for children, often correlating with higher levels of well-being. Conversely, single-parent families might face more challenges due to limited resources and less social support, potentially affecting the well-being of family members. However, we did not find any significant difference in the well-being scores between the “two-parent” and “single parent and others” groups. This is somewhat contradictory to Zajonc’s findings of “single and two parent situations affect the cognitive, intellectual, temperamental and linguistic environment in which individuals develop” (Zajonc, 1976). Several reasons could account for this result. First, we assume that participants from single-parent or other non-traditional family structures may have developed a higher level of resilience and adaptability due to facing greater adversities. This resilience might contribute positively to their sense of well-being. Secondly, they may have a different appreciation and sensitivity toward happiness, which enables them to maintain wellbeing levels comparable to their counterparts from traditional two-parent families, despite potential disparities in other dimensions. In addition, social and cultural contexts may also influence the sense of well-being. For instance, the Chinese society may offer a supportive system and set of values distinct from Western societies, which could mitigate the impact of family structure differences on an individual’s well-being. It was also found that the wellbeing literacy of the Chinese military academy cadets did not differ significantly from whether or not they were the only child, which is also different from existing studies (Jones and Adamson, 1987; Lambrecht et al., 2019) from the perspective of language development. Chinese military academy cadets might possess unique psychological and social traits, such as heightened discipline and a sense of belonging to a group, which could diminish the impact of family background on their well-being. The study suggests that although family structure may impact an individual’s development and sense of well-being, other factors such as the individual’s cognitive style, assessments of happiness, emotional sensitivity, adaptability, and potential cultural and social variables, are also at play. Therefore, future research needs to explore these potential factors and their interactions more comprehensively in order to better understand the influence of family structure on well-being.

## 5 Limitations

Limitations of this study include that the study is basically cross-sectional using only self-reported data, which may lead to response bias. Second, the re-validation within the military academy is merely a starting point that acknowledges the distinctive context of military education. Ideally, subsequent research should indeed extend to include various populations within China to establish a more comprehensive understanding of the scale’s generalizability and applicability across different Chinese cultural contexts, thereby allowing for a broader application of well-being literacy assessments and interventions across the country. Furthermore, within the constraints of this particular study, we aimed to include as many females as conditions would permit to enhance representativeness. Even with a male-dominated sample, all statistical analysis was performed with an acute awareness of this limitation, and the findings are interpreted with caution, particularly regarding the generalizability across genders. Future studies could aim to balance gender representation more effectively, perhaps by including civilian educational institutions, to provide a more holistic view of the studied phenomenon and to understand the nuances that gender differences might reveal in well-being literacy.

## 6 Conclusion

The Chinese version of the Well-Lit 6 has been proved to be a valid and reliable measure to assess Chinese military academy cadets’ wellbeing literacy, and the theoretical structure of well-being among Chinese cadets is still distinctive and closely related to their gender, grade and parenting style.

## Data availability statement

The raw data supporting the conclusions of this article will be made available by the authors, without undue reservation.

## Ethics statement

The studies involving humans were approved by the Ethics Committee of Army Medical University. The studies were conducted in accordance with the local legislation and institutional requirements. The participants provided their written informed consent to participate in this study.

## Author contributions

ZJ: Methodology, Writing – original draft, Conceptualization. FZ: Writing – original draft, Supervision. FW: Formal analysis, Funding acquisition, Methodology, Writing – review & editing. GY: Funding acquisition, Writing – review & editing.
